# Contribution of macrophages to intracochlear tissue remodeling responses following cochlear implantation and neural survival

**DOI:** 10.21203/rs.3.rs-3065630/v1

**Published:** 2023-06-26

**Authors:** Muhammad Taifur Rahman, Brain J. Mostaert, Bryce Hunger, Utsow Saha, Alexander D. Claussen, Ibrahim Razu, Nasrin Farjana, Nashwaan Ali Khan, Sarah Coleman, Jackob Oleson, Jonathon Kirk, Hirose Keiko, Marlan R. Hansen

**Affiliations:** Department of Otolaryngology-Head and Neck Surgery, The University of Iowa, IA; Department of Otolaryngology-Head and Neck Surgery, The University of Iowa, IA; Department of Otolaryngology-Head and Neck Surgery, The University of Iowa, IA; Department of Otolaryngology-Head and Neck Surgery, The University of Iowa, IA; Department of Otolaryngology-Head and Neck Surgery, The University of Iowa, IA; Department of Otolaryngology-Head and Neck Surgery, The University of Iowa, IA; Department of Otolaryngology-Head and Neck Surgery, The University of Iowa, IA; Department of Otolaryngology-Head and Neck Surgery, The University of Iowa, IA; Department of Statistics, The University of Iowa, IA; Department of Statistics, The University of Iowa, IA; Cochlear Limited, Sydney; Department of Otolaryngology-Head and Neck Surgery, Washington University School of Medicine, St Louis, MO; Department of Otolaryngology-Head and Neck Surgery, The University of Iowa, IA

**Keywords:** Cochlear implant, foreign body response, fibrosis, biomaterials, inflammation

## Abstract

**Introduction:**

Cochlear implants (CIs) restore hearing to deafened patients. The foreign body response (FBR) following cochlear implantation (post-CI) comprises an infiltration of macrophages, other immune and non-immune cells, and fibrosis into the scala tympani; a space that is normally devoid of cells. This FBR is associated with negative effects on CI outcomes including increased electrode impedances and loss of residual acoustic hearing. This study investigates the extent to which macrophage depletion by an orally administered CSF-1R specific kinase (c-FMS) inhibitor, PLX-5622, modulates the tissue response to CI and neural health.

**Materials and methods:**

10–12-week-old CX3CR1+/GFP Thy1+/YFP mice on C57Bl6 background with normal hearing were fed chow containing 1200 mg/kg PLX5622 or control chow for the duration of the study. 7-days after starting the diet, 3-channel cochlear implants were implanted ear via the round window. Serial impedance and neural response telemetry (NRT) measurements were acquired throughout the study. Electric stimulation began 7 days post-CI until 28- days post-CI for 5 hrs/day, 5 days/week, with programming guided by NRT and behavioral responses. Cochleae harvested at 10-, 28- or 56-days post-CI were cryosectioned and labeled with antibody against α-smooth muscle actin (α-SMA) to identify myofibroblasts and quantify the fibrotic response. Using IMARIS image analysis software, the outlines of scala tympani, Rosenthal canal, modiolus and lateral wall for each turn were traced manually to measure region volume. Density of nuclei, CX3CR1+ macrophages, Thy1+ spiral ganglion neuron (SGN) numbers and ratio of volume of α-SMA+ space/volume of scala tympani were calculated.

**Results:**

Cochlear implantation in control diet subjects caused infiltration of cells, including macrophages, into the cochlea: this response was initially diffuse throughout the cochlea and later localized to the scala tympani of the basal turn by 56-days post-CI. Fibrosis was evident in the scala tympani adjacent to the electrode array. Mice fed PLX5622 chow showed reduced macrophage infiltration throughout the implanted cochleae across all timepoints. However, scala tympani fibrosis was not reduced relative to control diet subjects. Further, mice treated with PLX5622 showed increased electrode impedances compared to controls. Finally, treatment with PLX5622 decreased SGN survival in implanted and contralateral cochleae.

**Discussion:**

The data suggest that macrophages play an important role in modulating the intracochlear tissue response following CI and neural survival.

## 1. Introduction

Cochlear implants (CIs) provide auditory rehabilitation to individuals with moderate to severe sensorineural hearing loss. The device has undergone tremendous technological advancements to broaden the range of candidacy including those at the extremes of age, individuals with residual hearing or unilateral hearing loss, and patients with auditory neuropathy spectrum disorder.(Roche & Hansen, 2015) Improved surgical techniques have led to a reduction in insertion trauma and electrode array translocation.(Ishiyama, Ishiyama, Lopez, & Linthicum, 2019). While modern surgical techniques and biocompatible implant materials enable a high rate of long-term device function, an intracochlear tissue response in the form of a foreign body response (FBR) or hypersensitivity reaction to the CI electrode array has been widely documented (Nadol, Eddington, & Burgess, 2008; Noonan, Lopez, Ishiyama, & Ishiyama, 2020; Seyyedi & Nadol, 2014) primarily as a delayed phenomenon.(Foggia, Quevedo, & Hansen, 2019; Li, Somdas, Eddington, & Nadol, 2007) This tissue response largely occurs within the scala tympani, a space that is normally devoid of cells making it unique to most other instances of FBR that occur in cellular tissues. Rahman et al. recently reviewed the current understanding of the inflammatory FBR following cochlear implantation, its impact on implant function in human subjects and animal models, and the emerging mitigation strategies for this deleterious response following cochlear implantation. (Rahman et al., 2022)

Histopathologic studies on human cochleae from CI recipients reveal an intrascalar tissue response comprised of densely organized fibrotic tissue and new bone growth (Seyyedi & Nadol, 2014) with variable severity occurring in a majority of cases. (Nadol et al., 2008). This post-CI FBR is associated with significant detrimental consequences including loss of spiral ganglion neurons (SGNs), poorer auditory function (Ishiyama et al., 2019), late onset loss of residual low frequency acoustic hearing (Quesnel et al., 2016), increased electrode impedances (Shaul et al., 2019; Tykocinski, Cohen, & Cowan, 2005), loss of acoustic hearing (Scheperle et al., 2017), poor word recognition scores (Kamakura & Nadol, 2016), and, in rare cases, late onset device failure (Nadol et al., 2008). While the FBR to electrode array biomaterials appears universal in human cochleae, it is exacerbated by traumatic insertion (e.g., electrode array translocation from scala tympani into scala vestibuli or media or damage to the lateral wall of the scala tympani).

Similar to humans, histological evidence for post-CI FBR occurs in animal models and is accompanied by loss of residual hearing, hair cells and SGNs. (Rahman et al., 2022) Hearing loss occurs early after implantation in guinea pigs and is followed by a partial recovery, which may be limited by the FBR. (Zhang, Stark, & Reiss, 2015) (Shepherd, Matsushima, Millard, & Clark, 1991). A correlation between fibrous tissue growth and sensory hair cell loss has been shown in cat (Clark, Shute, Shepherd, & Carter, 1995), guinea pigs (O’Leary et al., 2013), and macaques (Shepherd, Clark, Xu, & Pyman, 1995). Likewise, SGN loss is associated with electrode insertion trauma and inflammation in hearing cats(Xu, Shepherd, Millard, & Clark, 1997). The electrically evoked compound action potential (ECAP) reflects the synchronous response of auditory nerve fibers upon electrical stimulation; ECAP amplitude growth function is used to assess the SGN population health. CI insertion trauma and the FBR appear to cause SGN dysfunction (Pfingst et al., 2015; Schvartz-Leyzac et al., 2020) and, correspondingly, fibrosis and new bone formation are correlated with elevated ECAP thresholds in guinea pigs. (Simoni et al., 2020) Further, a correlation between the extent of the FBR after cochlear implantation and electrical impedance changes has been documented in guinea pig(Wilk et al., 2016), kitten(Ni et al., 1992), cat (Clark et al., 1995; Xu et al., 1997), and macaque models (Shepherd et al., 1995).

Macrophages have been identified in implanted human cochleae using antibodies to the markers CD163, Iba1, and CD68 (O’Malley, Nadol, & McKenna, 2016). These have been shown to phagocytize platinum and silicone from the electrode arrays (Nadol, O’Malley, Burgess, & Galler, 2014). ‘Activated’ macrophage are present within the fibrotic sheath surrounding the electrode arrays (Okayasu, Quesnel, O’Malley, Kamakura, & Nadol, 2020) and demonstrate increased responses in cases of translocation of the electrode array and damage to lateral wall (Noonan et al., 2020). In the mouse model, monocyte/macrophage (F4/80 positive cells) infiltrate in the cochlea in an apparent biphasic pattern: an early (3 days post implantation) and late (14–28 days post-implantation) peaks.(Bas et al., 2015; Claussen et al., 2022) Macrophages can drive pro-inflammatory and pro-healing responses; their contribution to the unique FBR that develops within the cochlea remains unkown. Beyond cellular infiltration, a fibrotic tissue response develops within the scala tympani after CI, evident by deposition of alpha-SMA positive cells and type I collagen.(Bas et al., 2015)

Given the deleterious consequences of the FBR, various strategies have been explored to mitigate this response. One such approach is the use of systemic or locally delivered glucocorticoids such as dexamethasone. Compared with standard CIs, electrode arrays that elute dexamethasone decrease fibrosis, bone growth and electrical impedances, protect hair cells, and help preserve auditory function after implantation without affecting SGN density in guinea pigs. (Ahmadi et al., 2019; Bas et al., 2016; Liu et al., 2015; Simoni et al., 2020; Van De Water et al., 2010; Wilk et al., 2016) and non-human primates. (Manrique-Huarte et al., 2020). Beyond dexamethasone, other anti-inflammatory medications that have been used to mitigate the post CI FBR include etanercept (a tumor necrosis factor alpha receptor antagonist), lipoic acid, and others.(Rahman et al., 2022)

One limitation of these approaches is that they use non-specific immunosuppressive agents that impact on a variety of immune cell types and cytokines. While macrophages comprise a major immune cell type involved in FBR post CI (Claussen et al., 2022) other immune cell types including T and B lymphocytes, cytokines (CXCL1, IL-1β, TNF-α) cell adhesion molecules (ICAM-1), connective tissue growth factor (CTGF), tissue remodeling proteins (TGF-β, MMP2, MMP9) are also involved in the FBR post-CI. (Rahman et al., 2022) While non-selective anti-inflammatory drugs help mitigate the FBR post-CI and improve functional outcomes, these agents preclude investigation of the contribution of specific cells or cytokines to the FBR.

In this study we focused on determining the role of macrophages and the innate immune response to the FBR post-CI and to neural survival. To this end, we used the colony stimulating factor 1 receptor (CSF-1R) inhibitor, PLX-5622, to deplete macrophages in a CX3CR1^+/GFP^ reporter mouse model of cochlear implantation. CSF1R is activated by 2 ligands, colony-stimulating factor-1 (CSF-1) and interleukin-34 (IL-34), and plays a critical role in development of microglia and most tissue macrophages. (Stanley & Chitu, 2014). In selecting a CSF1R inhibitor, we considered several factors. First, the inhibitor needs to penetrate blood-labyrinth barrier and second, post-CI FBR is a chronic inflammatory condition where macrophages are involved for extended period. Considering these factors, a highly selective CSF1R inhibitor, PLX5622 (PLX), that can cross the blood brain barrier and allows extended elimination of macrophages was employed in this study (Spangenberg et al., 2019)

## 2. Materials and methods

### 2.1. Animals

All the experimental protocols on mice in this study were approved by the University of Iowa Institutional Animal Care and Use Committee. For this study, we used both male and female 8–12 week old CX3CR1^+/GFP^ Thy1^+/YFP^ mice (n = 29) on a C57BL/6J background in which macrophages express GFP(Jung et al., 2000) and spiral ganglion neurons express YFP(G. Feng et al., 2000). To maximize utilization of the available CX3CR1^+/GFP^ mice, some wildtype Thy1^+/+^ mice were used as well while no homozygous CX3CR1^GFP/GFP^ or Thy1^YFP/YFP^ subjects were used. Genotyping was performed for CX3CR1 and Thy1 using automated standard PCR of genomic DNA from tail samples, performed by Transnetyx genotyping services (https://www.transnetyx.com/)

### 2.2. PLX5622 administration

**Figure 1:**
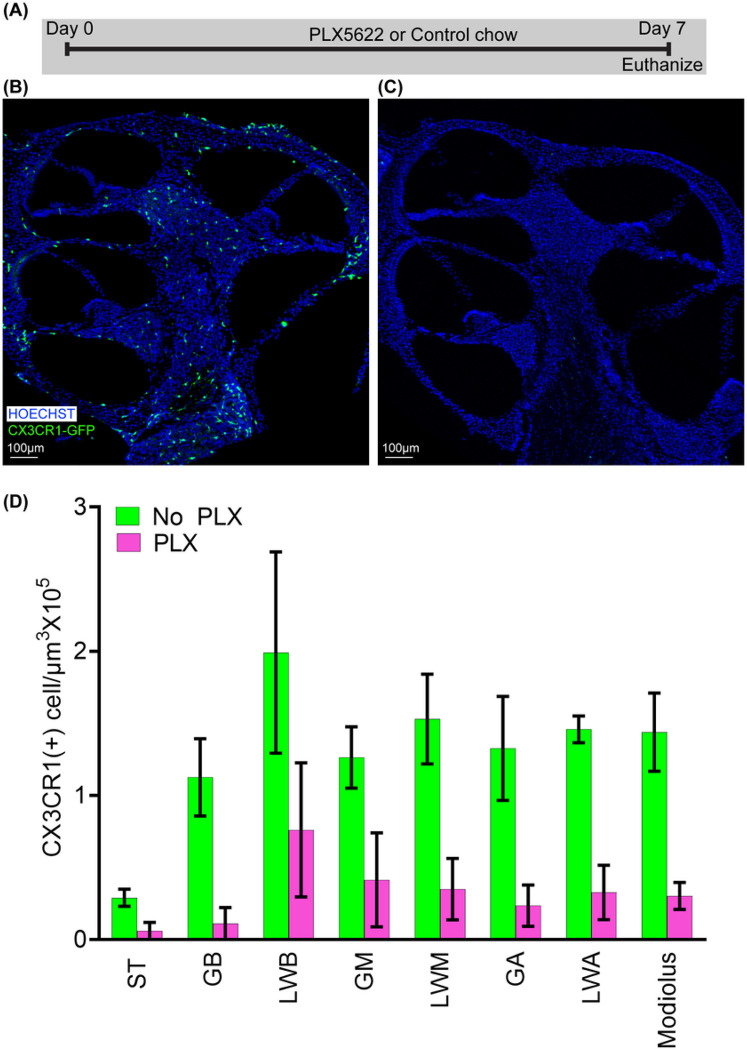
Depletion of Cochlear CX3CR1+ cells by oral administration of CSFR1 inhibitor, PLX5622. A. Study design for oral administration of CSFR1 inhibitor, PLX5622. Two groups of 10–12-week-old CX3CR1^+/GFP^ Thy1^+/YFP^ mice with C57B6 background were fed chow mixed with PLX5622 (PLX) or control chow (No PLX). At day 7, all mice were euthanized, labeled with Hoechst 3342 for histopathologic examination and confocal imaging. Confocal microscopy images showing CX3CR1-positive cells in (B) No-PLX and (C) PLX groups. D. Graphical representation of effect of 7 days of PLX-5622 treatment on cochlear resident CX3CR1-positive cells.

Macrophage depletion was achieved with PLX5622 diet to determine the role of macrophages following cochlear implantation. Preliminary experiments on non-implanted mice were performed to confirm macrophage depletion with oral PLX5622 administration. Specifically, we fed 2 groups of CX3CR1^+/GFP^ Thy1^+/YFP^ mice with chow with PLX5622 (PLX) or control chow (No PLX). PLX-5622 compounds were formulated in AIN-76A standard chow by Research Diets Inc. at 1200 ppm as described previously. Based on results from previous study, this dose is sufficient to deplete microglia in brain within 7 days (Dagher et al., 2015). Therefore, we tested whether this dose (1200ppm) and duration (7days) is sufficient to eliminate cochlear macrophages. At day 7, all mice were euthanized for histopathologic examination and confocal imaging. The timeline for this experiment is shown in Fig. 1 (Fig. 1a).

**Figure 2:**
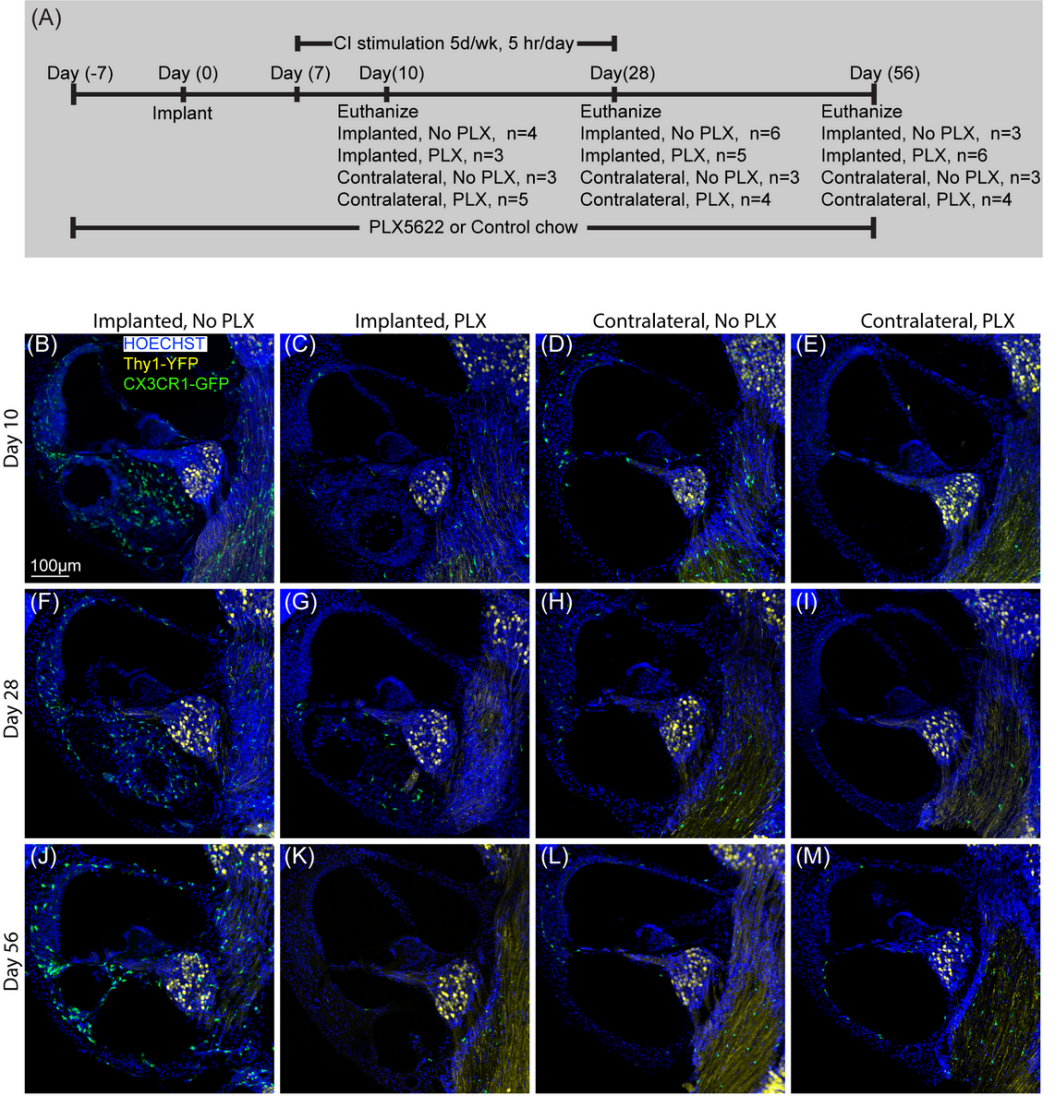
Cochlear implantation following elimination of cochlear macrophages. A. Study design for cochlear implantation in PLX5662 treated mice. Cochlear implantation was performed on 2 groups of CX3CR1^+/GFP^ Thy1^+/YFP^ mice: one group was fed on chow with 1200ppm of PLX-5622 (PLX) and the other group with control chow (No PLX) for 7 days. CI surgeries were performed on the left ear of both groups of mice after that (day 0). Following recovery from surgery, mice were continued with respective chow (PLX or No PLX) until they were sacrificed at 10-, 28- and 56-days post implantation. Starting on post-operative day 7, mice within stimulation cages, connected to the CI processor were stimulated for 5 h per day, 5 days a week. B-M: Fluorescent microscopic images of representative mid-modiolar, 30μm thick sections of the basal turn of the cochleae from respective groups. The following labels were used (Hoechst, blue), macrophages (CX3CR1-GFP, green) and neurons, (Thy1-YFP, yellow). In the presence of functional cochlear implant, macrophage (CX3CR1+ cells) infiltration into the cochlea appears to be sustained until the end of the study period (56 days post-CI). This was associated with infiltration of other cells labeled with nuclear labeling Hoechst.

After confirmation of cochlear macrophage depletion with PLX5622 chow in nonimplanted mouse cochlear implantation on 2 groups of CX3CR1^+/GFP^ Thy1^+/YFP^ mice. In PLX-5622 group (PLX), cochlear macrophages were depleted with oral administration of PLX5622 (1200ppm for 7 days). In ‘control’ (No PLX) group, CX3CR1^+/GFP^ Thy1^+/YFP^ mice were fed with ‘control chow’ for 7 days before CI surgery. On day 7, CI surgery was performed on both groups. Following recovery from surgery, mice from both groups were continued with respective chow (PLX or No PLX) until they were sacrificed at 10-, 28- and 56-days post implantation. The timeline for this experiment is presented in Fig. 2 (Fig. 2a)

### 2.3. Cochlear implantation

According to previously described technique, CI surgery was performed on left ears of mice (PLX or No PLX) through a round window approach with a custom 3 half-banded electrode cochlear implant (Cochlear Ltd., AUS) (Claussen et al., 2022; Claussen et al., 2019).

### 2.4. Impedance measurement, neural response telemetry (NRT) and chronic electrical stimulation

Impedance measurements, NRT (8th nerve electrically evoked compound action potential) and programming for chronic electrical stimulation were performed in Custom Sound EP 4.2 (Cochlear Ltd., AUS) according to previously published protocol. (Claussen et al., 2019). The Custom Sound programming software uses units of current level (CL) between 0 and 255, where 0 CL corresponds to 17.5 μA and 0.44 nC/phase, and 255 CL corresponds to 1750 μA and 43.75 nC/phase. Impedance measurements were performed for each separate electrode immediately prior to implantation. Immediately following surgery, impedance and NRT thresholds were measured and repeated at least weekly thereafter. Electrodes within compliance limits (defined as having electrical impedance ≤ 35kOhms) were shorted together during electrical stimulation using a software patch. The hardware system for electrical stimulation consisted of a modified rodent housing with a sliding tether connected to a CI emulator (CIC4 implant emulator, Cochlear Ltd., AUS) which was activated by interfacing the receiver coil with a commercial CI sound processor (Cochlear Ltd., AUS). Starting on post-operative day 7, mice placed within and connected to the aforementioned system were stimulated for 5 h per day, 5 days a week, programmed to 30CL below NRT threshold with a dynamic range of 1CL between threshold and comfort levels. (Claussen et al., 2019) MAPs were reprogrammed weekly based on changes in electrode function and/or NRT threshold. Electrical stimulation was provided through to 28 days postoperative. In this model and system electrode failures become more common beyond 28 days (Claussen et al., 2019) and it becomes difficult to provide consistent stimulation across each group.

### 2.5. Immunohistochemistry

Under anesthesia with ketamine (80mg/kg) and xylazine (10mg/kg), mice were exsanguinated with transcardial perfusion of ice-cold Phosphate Buffer Solution (PBS) followed by transcardial perfusion of 4% paraformaldehyde (PFA) at the final respective timepoint for each subject. Cochleae were harvested and were fixed overnight with 4% paraformaldehyde at 4°C in dark. Excess PFA was removed with PBS in a rotator overnight. Decalcification of cochlea was done in 0.1M EDTA (pH 7.5) solution on a rotator that is changed every day for 3–5 days. After decalcification, cochlea washed with PBS 3 times, 10 minutes each time. Decalcified cochleae were cryoprotected using serial concentration of sucrose solutions starting at 10% sucrose solutions and increasing concentration by 10% every hour, finally reaching 30%. Cryoprotected mice cochleae were stored at −20 C until sectioned. Cochlea infused with O.C.T. embedding medium (Tissue-TEK) were then mounted to the stage of sliding block microtome (American Optical 860) stage with O.C.T. and dry ice. Mounted cochleae were sectioned parallel to the mid-modiolar plane at 30μm thickness, sections were placed on Fisher Superfrost slides, and stored at − 20°C until immunolabeling was performed. For immunolabeling, slides were first warmed to room temperature (~ 20–22°C), washed (3 × 5 min each wash) with ‘washing buffer’ containing PBS, 0.03% Triton X-100 and 0.1% Tween-20. Then samples were permeabilized and blocked in ‘blocking buffer’ (0.03% Triton X-100 and 0.1% Tween-20, 1% bovine serum albumin (RPI, CAS#9048-46-8) and 0.02% sodium azide (Sigma, catalog #S2002) in PBS for 2 hours. Following blocking, sections on slides were incubated in primary antibody (Alpha-smooth muscle actin monoclonal antibody, 1A4, eBioscience^™^, Catalog# 14-9760-82 in ‘blocking buffer’ overnight (~ 16 h) at 4°C. After primary antibody application, sections were washed (3 × 5 min) in ‘washing buffer’, then incubated in blocking buffer containing secondary antibodies (Alexa Fluor conjugates, 1:400, Invitrogen) for 2-hrs at room temperature. Sections were then washed 3 × 5 min in ‘washing buffer’. Nuclei were stained with Hoechst 3342 (10 μg/ml in PBS, Sigma) for 20 min at room temperature, followed by washing with ‘washing buffer’ (3 × 5 min) and cover slipped with Fluoro-Gel Mounting Medium with Tris Buffer (catalog #17985-10, Electron Microscopy Sciences).

### 2.6. Imaging and image analysis

For histological analysis of nuclei, macrophage, neurons, and fibrosis, 30μm thick sections from cochleae (implanted or contralateral) were immunolabeled, as described above, to visualize macrophage (CX3CR1-eGFP) neurons (Thy1-eYFP), nuclei (Hoechst 3342) and fibrotic tissue (anti-SMA antibody with Alexa 568 conjugated secondary antibody). Fluorescently labeled cochlear sections were imaged on a Leica Stellaris 5 confocal system using a 20x (0.70 NA) objective, 0.75x digital zoom, z axis-spacing of 1 μm and keeping exposure/gain settings constant throughout the experiment. Cochlear mid-modiolar sections were labeled for quantification of different cell types and tissues with the data from 3 adjacent sections averaged (“n” refers to number of subjects). Image analysis was performed in IMARIS (Oxford Instruments, UK) image analysis software, cell counts, and quantitation of volumetric analyses were done on maximum intensity z-projections of 3D confocal image stacks. First, the outline of Rosenthal’s canal (RC) and the lateral wall in the basal, middle and apical turns, the basal turn scala tympani and modiolus were traced to measure the volume of each area. The number of macrophages (CX3CR1^+/GFP^), neurons (Thy1^+/YFP^) and nuclei (Hoechst 3342) were counted using automated counting system in IMARIS image analysis software. The density of nuclei, macrophage and neurons were the calculated per 10^5^ μm^3^. Cell counting was restrained to the requirement of co-localized nuclei (i.e., one neuron or macrophage counted per single co-localizing nuclei and reporter marker of interest). The fibrotic response was assessed by volumetric quantification of SMA in the basal scala tympani in midmodiolar sections referenced to the volume of scala tympani. For histological analyses, we defined cochlear location by half-turn increments proceeding from the base to the apex, as previously described. (Kopelovich, Cagaanan, Miller, Abbas, & Green, 2013). To allow quantitative comparison between experimental and control sections, image capture was performed using identical settings for imaging. Personnel performing cell counting, volumetric analyses, and other analyses were blind to the experimental conditions.

### 2.7. Statistical analyses

Statistical analyses for impedance measurements, counts of immune cells, nuclei, neurons, and volume of fibrotic tissue within scala tympani were performed using R version 4.3.0 (2023-04-21)(“R Core Team,” 2021). Specific comparisons that were made are described in respective figure legends. General linear models were fit to assess main effects of group, day and their interaction, except in impedance data where non-linear trends were expected respective to time and a linear mixed model was fit. Shapiro-Wilk or D’Agostino-Pearson test was used to determine normality of data. If model assumptions were not met, simpler models that excluded outlier groups were fit for parametric tests. For parametric data, pairwise comparisons of the least square means were made with Tukey adjustment for multiple comparisons. Additionally, non-parametric tests (e.g., Kruskal-Wallis Test) were performed Using Wilcoxon Rank scores to compare all groups in data that were not normally distributed. Results of the statistical analysis are included in the text; as a regression analysis approach was used where comparisons are made on the regression models, no statistical annotations are included on the figures. Significance was defined as p < 0.05).

## 3. Results

### 3.1. PLX5622 depletes cochlear resident macrophages

Figure 1 presents representative mid-modiolar sections of cochleae from CX3CR1^+/GFP^ reporter mice following 7 days of administration of PLX5622 (PLX) or control chow (No PLX). In cochleae from No PLX mice, CX3CR1-positive cells exist in specific regions: scala tympani of base of cochlea, spiral ganglion, modiolus, and lateral wall (base, middle and apex). Seven days of oral treatment with chow containing 1200 ppm of PLX-5622 nearly eliminates (~ 80%) these resident CX3CR1-positive macrophages (unpaired t test, p = 0.0001). Going forward we used this protocol to deplete resident macrophages prior to cochlear implantation.

### 3.2. PLX5622 reduces macrophage infiltration post-CI

**Figure 3:**
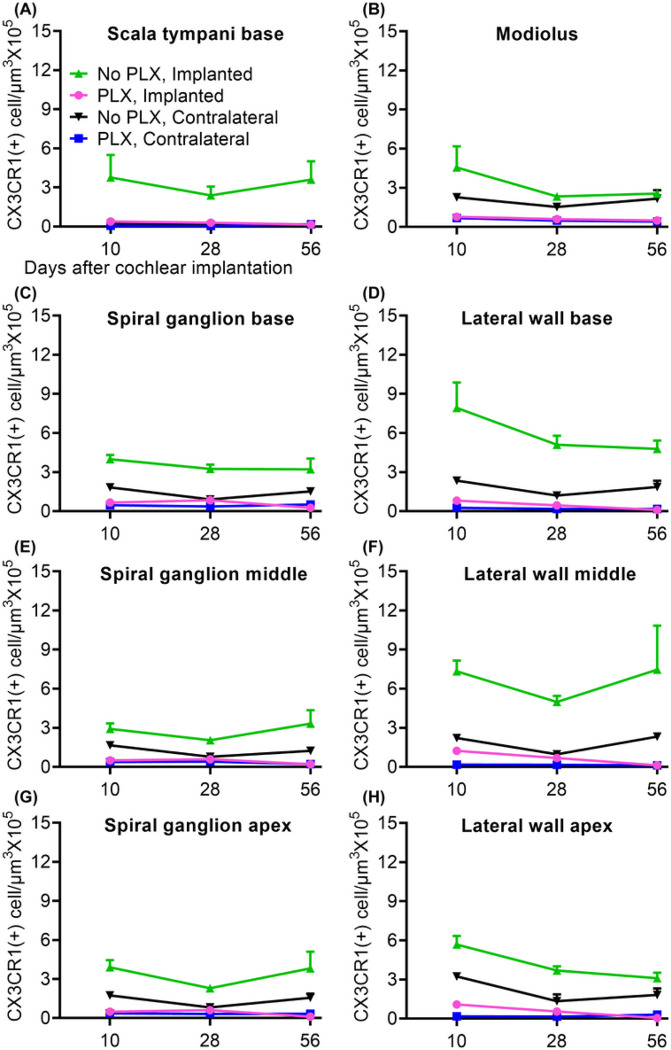
Quantification of CX3CR1+ macrophage density in cochlea following cochlear implantation. Cochlear implantation was performed in CX3CR1^+/GFP^ Thy1^+/YFP^ mice, fed on chow with 1200ppm of PLX-5622 (PLX) or control chow (No PLX) for 7 days. Following surgery, mice were continued with respective chow (PLX or No PLX). Starting on post-operative day 7, mice within stimulation cages, connected to the CI processor were stimulated for 5 h per day, 5 days a week. Mice were euthanized at 10-, 28- and 56-days post-CI and imaged as in Figure 3. Image analysis was performed in IMARIS image analysis software. In 30μm thick midmodiolar sections, CX3CR1+ macrophage cells were counted on maximum intensity z-projections of 3D confocal image stacks. The outline of Rosenthal’s canal (RC) and lateral wall at base, middle and apex of cochlea, scala tympani of the base of the cochlea and modiolus were traced and volume of each area was measured. CX3CR1+ macrophages were counted using automated counting system in IMARIS image analysis software aided by custom made macros. The density of macrophages with visible, Hoechst+ nuclei was calculated per 10^5^ μm^3^ in each area mentioned. Values derived from every region of cochlea for an individual animal were averaged together from 3 sections; “n” is the total number of mice used in the study. Error bars indicate SEM.

We next examined whether PLX5622 also reduces the CX3CR1-positive cochlear macrophage population following cochlear implantation. After 7 days treatment with either PLX-5622 chow (PLX) or control chow (No PLX), left cochleae of CX3CR1^GFP/+^ Thy1^YFP/+^ mice were implanted and stimulated for up to 28 days post-CI while right cochleae served as unimplanted controls. Animals were euthanized at 10, 28, or 56-days post-CI. Representative mid-modiolar sections across groups and timepoints are seen in Fig. 2. Figure 3 demonstrates the quantification of CX3CR1-positive cell density in different regions of cochlea across the study period. In the No PLX unimplanted cochleae, CX3CR1 + cells were dispersed throughout the cochlea including a very low density along the margin of the scala tympani of the base. These cells demonstrate classical ramified morphology of tissue macrophages. Compared to the contralateral side, No PLX implanted cochleae showed significant infiltration of CX3CR1-positive cells throughout the cochlea (Fig. 2 and Fig. 3). While most of the infiltrating CX3CR1 + cells exhibited a ramified morphology in the No PLX implanted cochleae, a small subset of ameboid CX3CR1-positive cells were observed in the scala tympani. A major influx of CX3CR1-positive cells within the modiolus, the scala tympani, spiral ganglion, lateral wall of base middle and apical turn was observed following implantation in No PLX (Kruskal-Wallis Test on Wilcoxon Rank scores, p < 0.0001 comparing all groups, a parametric model could not be fit to include No PLX implanted) as shown in Fig. 3. Within the specified period (10 through 56 days post-CI), time following cochlear implantation does not significantly affect the density of CX3CR1-positive cells (No effect of day in general linear model in all cases, p > 0.05) except in the lateral wall of the base, where a main effect of day was seen (p = .0013) with pairwise comparisons on the least squares means with Tukey adjustment showing day 10 macrophage density was significantly different than day 28 (p = 0.0016) and 56 (p = 0.0096), with no difference between day 28 and 56 (p = 0.7202) (Fig. 3).

Orally administered PLX5622 effectively reduced the resident macrophage population throughout the cochlea for the study duration in unimplanted cochleae compared to No PLX (pairwise comparisons of No PLX and PLX unimplanted groups on least squares mean effect of group using Tukey adjustment, p < 0.05 in all areas except scala tympani of base, p = 0.4295, where little to no macrophage infiltration was seen in either group). Likewise, oral administration of PLX5622 reduced the infiltration of CX3CR1-positive cells throughout implanted cochlea at all timepoints compared to No PLX implanted (Kruskal-Wallis Test on Wilcoxon Rank scores of non-parametric data, p < 0.0001 in all cases, a parametric model could not be fit to include No PLX implanted) (Fig. 3).

### 3.3. PLX5622 does not reduce cellular density within cochlea post-CI

**Figure 4:**
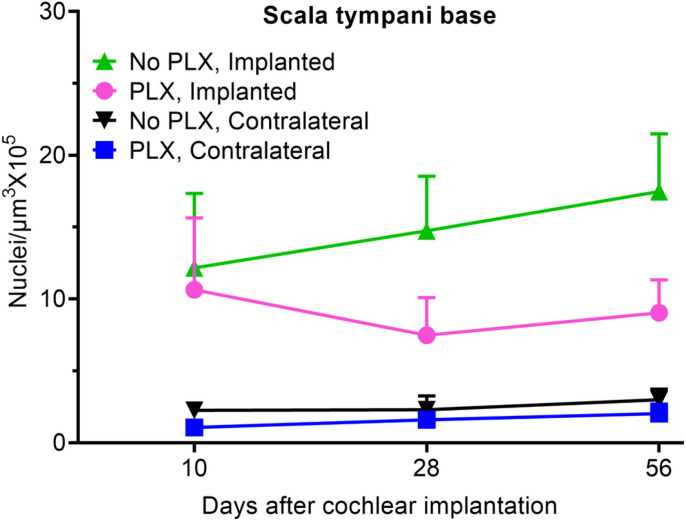
Quantification of cellular density into scala tympani of the base of cochlea following cochlear implantation. Cochlear implantation was performed in CX3CR1^+/GFP^ Thy1^+/YFP^ mice, fed on chow with 1200ppm of PLX-5622 (PLX) or control chow (No PLX) for 7 days. Treatment with respective diets and electrical stimulation was continued until the desired endpoints (10, 28 or 56 days). Nuclei were labeled with Hoechst 3342 in 30μm thick mid-modiolar sections. Image analysis was performed in IMARIS image analysis software. Hoechst+ cells were counted on maximum intensity z-projections of 3D confocal image stacks in scala tympani of the base of the cochlea. Volume of scala tympani and nuclear counts were made using automated counting system in IMARIS image analysis software aided by custom made macros. Nuclear density (Hoechst+ cells) was calculated per 10^5^ μm^3^. An average of 3 sections per animal was taken with ‘n’ being the number of animals. Error bars indicate SEM.

Cochlear implantation resulted in increased density of Hoechst + cells into cochlea in PLX and No PLX groups relative to unimplanted groups, shown histologically in Fig. 2 and quantified in Fig. 4 and Supplementary Fig. 1. Time following cochlear implantation (10 through 56-day post-CI) does not significantly affect cellular density (No effect of day in the parametric model including No PLX and PLX implanted groups, p = 0.8304). A trend of reduced cellular density was seen in the PLX implanted compared to the No PLX implanted approached but this did not reach statistical significance (No effect of group in the parametric model including No PLX and PLX implanted groups, p = 0.0829). As the data were not normally distributed, we used additional non-parametric testing on Wilxocon Rank Scores to assess all 4 groups. The Kruskal-Wallis Test was significant p < .0001, suggesting a difference between groups in scala tympani nucleus density in the basal turn (Fig. 2 and Fig. 4).

### 3.4. PLX5622 does not affect fibrosis post-CI

**Figure 5:**
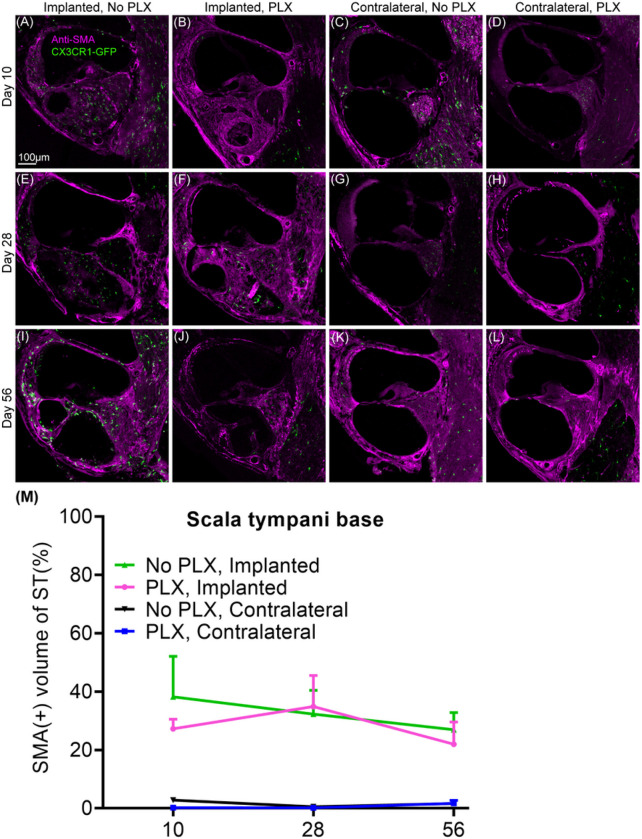
Quantification of SMA+ tissue within scala tympani of the base of the cochlea following cochlear implantation. Following 7-day feeding on chow with 1200ppm of PLX-5622 (PLX) or control chow (No PLX), cochlear implantation was performed in CX3CR1^+/GFP^ Thy1^+/YFP^ mice, Respective diets were resumed following recovery from surgery. Electrical stimulation was continued until 28-day post-CI. Mice were euthanized at the desired endpoints (10, 28 or 56 days). Following euthanasia, 30μm midmodiolar sections were labeled with anti-alpha SMA antibodies. The volume of the scala tympani and SMA+ tissue volumes were measured using IMARIS image analysis software. Fibrosis was measured by dividing the volume of SMA+ tissue by volume of scala tympani, expressed in % volume. Error bars indicate SEM.

Following cochlear implantation in PLX and No PLX mice, SMA + fibrotic tissue grows into the scala tympani of base of the cochlea adjacent to the electrode array (Fig. 5). Fibrosis is seen at 10 days post-CI and is maintained throughout the 56-day post-CI timepoint and time following cochlear implantation (10 through 56 days post-CI) does not significantly affect the volume of the fibrotic response (No effect of day in parametric model including PLX and No PLX implanted groups, p = 0.5975). Unlike the macrophage and cellular infiltration, the volume of SMA + fibrotic tissue in basal turn was not affected by PLX5622 treatment (No effect of group in parametric model including PLX and No PLX implanted groups, p = 0.5975) (Fig. 5).

### 3.5. PLX5622 increases electrode impedance

**Figure 6:**
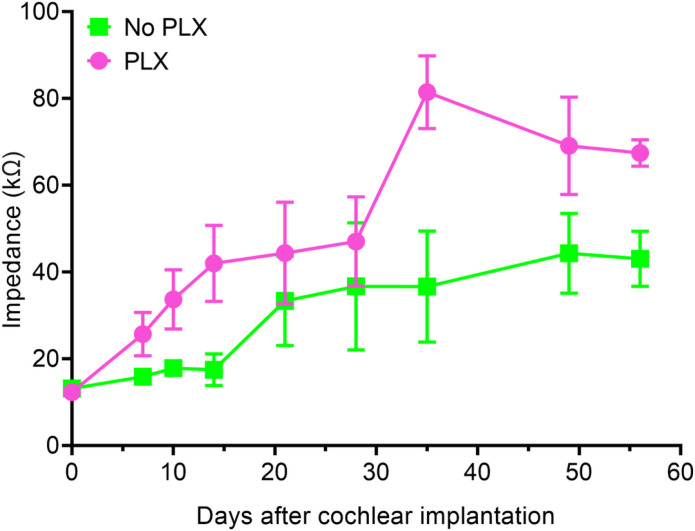
Electrode impedance following cochlear implantation plotted over time. Lines represent mean impedance values across all functional electrodes (without open circuit) at the representative timepoints for the No PLX (green line) and PLX groups (pink line). Error bars indicate SEM.

Figure 6 shows mean electrode impedance values for No PLX and PLX groups overtime for active electrodes (i.e., electrodes with an open-circuit denoting hardware failure were excluded). The trends in impedance growth over time for PLX and No PLX groups appeared non-linear, thus we used a linear mixed model using the square root of days. The PLX and No PLX groups showed similar baseline electrode impedance values at peri-operative baseline testing (linear mixed model, day 0 y-intercept, p = 0.9341). Both PLX (slope = 8.47 kOhm/square root of days p < 0.0001) and No PLX (slope = 4.05 kOhm/square root of days p = 0.0018) showed statistically significant increases in impedance over time, with the rate of increase in the PLX group being greater (group × square root of day interaction, p = 0.0067). We used contrasts (Kenward-Roger degrees of freedom and a Bonferroni alpha level correction) from the linear mixed model to assess at what point the two groups diverged in impedance growth over time. We observed that the PLX impedance values began to significantly differ from the No-PLX between day 14 and 21 and persist to 56 days. (p = 0.06 and 0.02 at 14- and 21-days post-CI, respectively; Kenward-Roger degrees of freedom and Bonferroni alpha level correction). 21-days post-CI and onward, electrical impedance in PLX group was consistently higher compared to the No PLX group (p = 0.0056–0.0104; Kenward-Roger degrees of freedom and Bonferroni alpha level correction).

### 3.6. PLX5622 causes SGN degeneration

**Figure 7:**
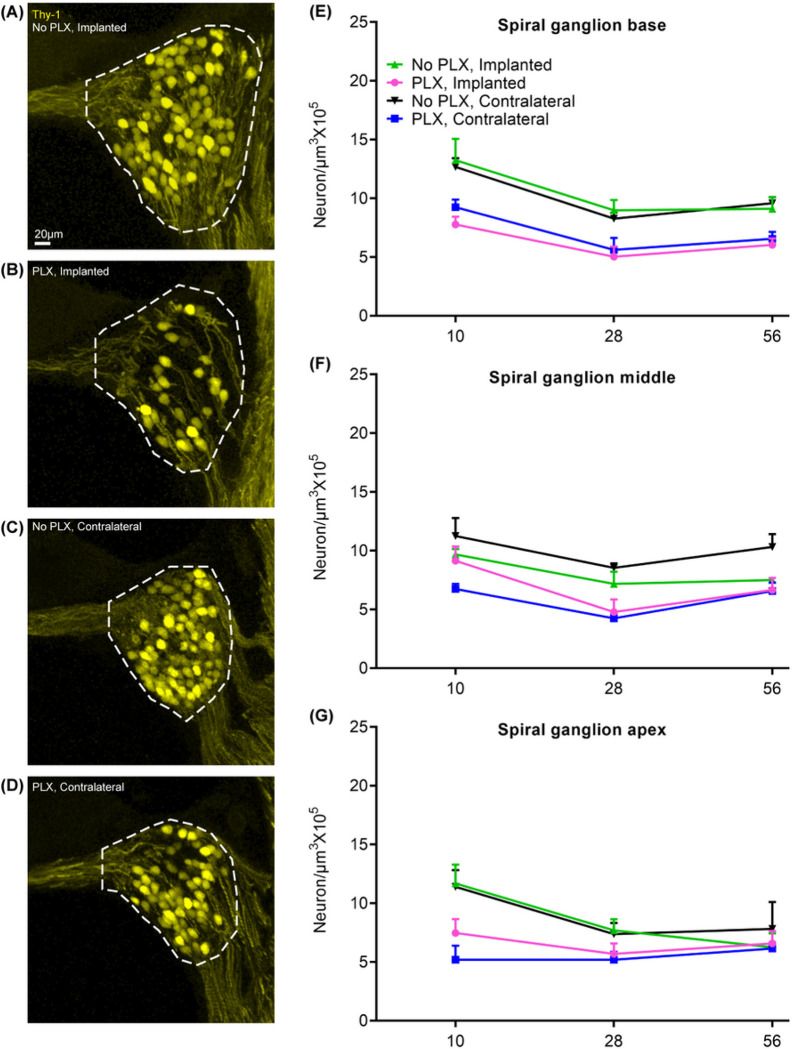
Quantification of spiral ganglion neuron density following cochlear implantation. 7-day feeding on chow with 1200ppm of PLX-5622 (PLX) or control chow (No PLX) was followed by cochlear implantation in CX3CR1^+/GFP^ Thy1^+/YFP^ mice. After recovery from surgical anesthesia, respective diets were resumed. Electrical stimulation was done as described before. Cochlea harvested at desired endpoints (10, 28 or 56 days) were sectioned at 30μm thickness. After measurement of spiral ganglia volume and quantification of spiral ganglion neurons, SGN density was calculated by dividing the SGN count by the volume and expressed as per 10^5^ μm^3^. Density in 3 sections per animal was averaged. Number of the animals is considered the ‘n’ for this experiment. Error bars indicate SEM.

Figure 7 shows mean density of Thy1^YFP^ + SGNs for the ‘No PLX’ and ‘PLX’ groups overtime. First, we considered the change in SGN density over time in the base (significant effect of day in parametric model, p < 0.0001). In the spiral ganglion of the base of the cochlea, SGN density at day 28 is significantly lower than that of day 10 (pairwise comparison on least squares means for effect of day with Tukey adjustment, p < .0001). The difference between day 10 and 56 was also significant (pairwise comparison on least squares means for effect of day with Tukey adjustment, p = .0011). The difference between day 28 and day 56 was non-significant. Similarly, SGN degeneration is observed in middle turn of cochlea from day 10 to day 28 (significant effect of day in parametric model, p = 0.0004; pairwise comparison on least squares means for effect of day with Tukey adjustment, p = .0003). In the apical turn, density at day 28 is significantly lower from day 10 (significant effect of day in parametric model, p = 0.0120; pairwise comparison on least squares means for effect of day with Tukey adjustment, p = 0.0148) The difference between day 10 and 56 in apical neuron density was marginally significant (p = 0.0503), but the difference between day 28 and day 56 was non-significant (p = 0.96). These experiments are done on mice with B6 background and suggest that significant SGN degeneration happens in these mice between 3 and 4 months of age. Next, we considered the effect of cochlear implantation on SGN density. Following cochlear implantation, we did not observe evidence for SGN degeneration in cochleae compared to respective PLX and No PLX unimplanted groups (significant effect of group in parametric model of apical, middle and basal neuron density, p = 0.0001–0.0051; pairwise comparison on least squares means for effect of day with Tukey adjustment, p > 0.05).

We also considered whether PLX treatment causes degeneration of SGNs in the unimplanted condition. Compared to ‘No PLX mice, in ‘PLX mice, SGN density was significantly lower in contralateral (unimplanted) cochlea at the basal turn (p = .0053), middle turn (p = .0002) and apical turn (p = .0148) (significant effect of group in parametric model of apical, middle and basal neuron density, p = 0.0001–0.0051; pairwise comparison on least squares means for effect of day with Tukey adjustment). We further analyzed the impact of PLX treatment in implanted cochlea. In implanted cochlea, PLX treatment is associated with SGN degeneration in the base only (p < .0001 significant effect of group in parametric model apical, middle and basal neuron density, p = 0.0001–0.0051; pairwise comparison on least squares means for effect of day with Tukey adjustment). After looking at pairwise comparisons in the group × day interaction, we found that the only significant differences between treatment groups are in day 10 and 28. At day 10 post-CI, the mean for PLX-CI is significantly lower than no-PLX CI (p = 0.0202) and at day 28, PLX CI is significantly lower than No PLX CI (p = .0487); pairwise comparison on least squares means for day × group interaction with Tukey adjustment. Thus, PLX5622 administration was associated with SGN degeneration irrespective of cochlear implantation status.

## 4. Discussion

Our data suggest that PLX5622, a specific inhibitor of CSF1R, at a dose of 1200 mg/kg for 7 days eliminated almost all cochlear mononuclear phagocytes. Following cochlear implantation, a cellular infiltration, including macrophages, with fibrotic tissue deposition occurs adjacent to the electrode array in the basal scala tympani and was associated with increased electrode impedance. When cochlear implantation was performed in mice with ongoing PLX 5622 macrophage depletion, cellular infiltration (including macrophage infiltration) was inhibited but the volume of fibrotic response was not. Electrical impedance following cochlear implantation trended higher in PLX5622 treated group. Moreover, PLX5622 treatment was associated with degeneration of SGNs in base of the cochlea independent of cochlear implantation.

With short term (7 days) administration of PLX5622 at a dose of 1200 mg/kg for 7 days, most CX3CR1-positive cells can be depleted from cochlea. This dosage is also sufficient to deplete brain microglia population. (Dagher et al., 2015) It has been shown previously that CSF1R inhibition by PLX5622 is not microglia specific; it can affect other mononuclear phagocyte populations (monocyte, macrophage, and dendritic cells) as well. (Lei et al., 2020) Although there is preliminary evidence of a cochlear microglia population, their relative abundance compared to other mononuclear phagocytes like macrophages and dendritic cells has not been established. (Seigel, Manohar, Bai, Ding, & Salvi, 2017) CX3CR1 is expressed in all types of mononuclear phagocytes.(J. Feng et al., 2022; Koscso et al., 2020; Wolf, Yona, Kim, & Jung, 2013). Here we observed that most, but not all, cochlear CX3CR1 + cells were depleted with short (or even long term) treatment with PLX5622, suggesting differing susceptibility among mononuclear phagocytes. The relative susceptibility of individual types of mononuclear phagocytes to CSF1R inhibition by PLX5622 is yet to be determined as are dose-specific effects. Our data also support the previously published literature showing that sustained treatment with PLX5622 in CX3CR1^GFP/+^ mice results in significant elimination of resident macrophages (~94%) without causing elevation of ABR threshold. (Manickam et al., 2023) This study also suggests that CSF1R is expressed on the cochlear CX3CR1 + cells explaining the depletion of CX3CR1 + cells with CSF1R inhibitor PLX5622. (Manickam et al., 2023)

PLX5622 not only depleted resident CX3CR1 + cells prior to cochlear implantation, it also caused sustained depletion of the infiltrating CX3CR1 + cells after placement of the electrode array. To the best of our knowledge, this is the first study to explore the role of CSF1R inhibition in a cochlear implant model. Studies on brain implants demonstrated similar effects on brain microglial population. (Sharon, Jankowski, Shmoel, Erez, & Spira, 2021) They have shown that although PLX5622 treatment depletes microglia from the rat brain, astrocytes encapsulate the neuro-implant suggesting that microglia are redundant for this FBR in the brain. The reduction in cellular density in the scala tympani of PLX treated mice following CI might be a direct effect of elimination of resident and infiltrating macrophage population. Also, in the spiral ganglia, we observed degeneration of SGNs that can contribute to the decline in cellular density within spiral ganglia. Moreover, macrophages also secrete growth and angiogenic factors. (Anderson, Rodriguez, & Chang, 2008) Thus, elimination of macrophages could indirectly reduce cellularity by decreasing cell proliferation and angiogenesis. Although macrophages are widely viewed as master regulators of the FBR to biomaterials, other innate and adaptive immune cells including T and B lymphocytes and mast cells contribute to these tissue responses. (Anderson et al., 2008) In the cochlea, the FBR to the implanted electrode array occurs in a unique environment in the scala tympani that is otherwise devoid of cells. While the role of these other immune cells has not been studied intensively in the cochlea, it has been documented that T and B lymphocytes infiltrate the cochlea following cochlear implantation. (Nadol et al., 2014) A wide range of cytokines can be secreted by activated macrophages; these include: Interleukins (e.g., IL-1, IL-4, IL-5, IL-6, IL-8, IL-10, IL-12, IL-18), TNF-α, and TGF-β.(Anderson et al., 2008) Many of these cytokines act as chemotactic factors for other immune cells. It is possible that depletion of resident and infiltrating macrophages by PLX5622 impacts recruitment of other immune cells and thus overall cellular infiltration of the scala tympani following CI.

One significant finding of these experiments is that the fibrotic response, as measured by anti-αSMA immunolabeling, was not significantly reduced by PLX5622 treatment. These results mirror other studies that explored the role of CSF1R inhibition with PLX5622 on the FBR to neuro-implants in the brain. (Sharon et al., 2021; Sharon, Jankowski, Shmoel, Erez, & Spira, 2023) Sharon et al. showed that PLX5622, depletes microglia in rat brain (Sharon et al., 2021), however, it does not inhibit the astrocyte response encapsulating the neural implant. (Sharon et al., 2021) These results with cochlear and neural implants are in sharp contrast with findings in non-neural tissue following implantation of biomaterials. Doloff et al. demonstrated that following implantation of biomaterials in non-neural tissue, elimination of macrophages with CSF1R inhibitor effectively suppressed the fibrotic response. (Doloff et al., 2017) There are several plausible explanations for this difference between neural and non-neural tissue including: 1. The pathophysiology of the FBR in neural tissue differs from that in non-neural tissue, 2. The implanted biomaterials were different in cases of implantation in non-neural tissue and elicited different, material specific types of FBR, and 3. The pharmacological agents that were used to deplete macrophages are different from what has been used in the neural tissue and had a different impact on fibrotic response.

Following CI, there is a gradual rise in electrode impedances consistent with an evolving tissue response. PLX 5622 treatment lead to a more rapid rise in electrical impedance compared to No PLX. As PLX5622 treatment reduces cellular infiltration into cochlea, it appears that reducing cellular infiltration alone is not sufficient to prevent the rise in electrical impedance associated with the FBR. Further the extent of fibrosis, as measured by anti-αSMA immunolabeling, is not affected by PLX5622 treatment suggesting that the fibrotic tissue might be the factor maintaining the high electrical impedance in PLX5622 treated implanted cochlea. Moreover, electrode impedances in mice treated with PLX5622 rose more rapidly than the impedances in control mice raising the possibility that there are functional differences in the nature of the fibrotic response in the absence of macrophages. Post-implantation cochlear fibrosis is often accompanied by neo-ossification in humans and mice. The current study methods employed decalcification for histological preparation, prohibiting assessments of cochlear neo-ossification after implantation. This aspect is important for future studies, as CSF1R inhibition is associated with alterations in osteoclast activity that could impact post-CI neo-ossification and differentially effect electrode impedance compared to the less dense, non-mineralized fibrotic tissue. (Brun et al., 2020)

Degeneration of SGNs in the base of the cochlea with PLX5622 treatment is noteworthy. The role of cochlear macrophages in the protection of SGNs depends on the model of cochlear insult. In a mouse model of selective hair cell destruction, fractalkine mediated infiltration of CX3CR1 + mononuclear cells protect SGNs from degeneration. (Kaur et al., 2015) Conversely, anti-inflammatory therapy with ibuprofen or dexamethasone has been shown to suppress the infiltration of macrophages in the spiral ganglion following aminoglycoside induced hair cell loss in rat model; this suppression of macrophage infiltration is associated with SGN protection. (Rahman et al., 2023).

Macrophage infiltration can be associated with protection or degeneration of SGNs depending on context and on chemokine receptor expression. CX3CR1 receptor deletion (CX3CR1KO) induces a distinct phenotype when compared to depletion of CX3CR1 expressing cells, as we demonstrate here with PLX5622. One potential explanation is that macrophages play diverse roles in SGN protection in deafening models: the fractalkine pathway is involved in SGN protection, whereas macrophages are involved in additional mechanisms that contribute to SGN degeneration. Therefore, selective inhibition of fractalkine is neurotoxic whereas non-selective inhibition of inflammation provides neuroprotection following deafening. In a noise-induced cochlear synaptopathy model, macrophages promote synapse regeneration. (Manickam et al., 2023) In the present study, macrophage infiltration into the spiral ganglia following cochlear implantation itself does not appear to cause SGN degeneration. We observed SGN degeneration in the base of the cochlea following PLX5622 treatment independent of cochlear implant surgery. This observation suggests a general protective role of macrophages for SGNs. However, there is an important confounder that makes the interpretation of our data more complex. Our experiments were done on mice with C57B6 background, whereas other deafening and synaptopathy experiments were performed on CBA/J mice and rats. C57B6 mice exhibit early onset hearing loss and SGN loss that is not observed in CBA/J mice or rats. (Kane et al., 2012) Importantly, the C57/B6 background mimics hearing loss patterns seen in many human CI candidates with post-lingual onset of high-frequency hearing loss. (Jensen et al., 2022) One plausible explanation for our results is that macrophages play a protective role for SGNs in C57/B6 mice and in the absence of macrophages, early onset SGN degeneration is accelerated. Experiments inhibiting CSF1R with PLX5622 in mice with CBA/J background can provide additional insights into this issue.

We would like to mention a potential limitation of the method of SGN quantification that we used. We observed variation in Thy1-driven YFP expression among the SGN population. Therefore, use of Thy1-reporter expression as a marker for SGN might present issues with reliability. Moreover, the sensitivity of Thy1-reporter as a marker for SGN is not currently known. These present findings are relevant to current efforts to develop pharmacologic based therapies to mitigate of CI insertion trauma, as the effect of a dexamethasone eluting electrode arrays (NCT04750642, NCT04450290) on macrophage suppression and subsequent SGN preservation or degeneration is not yet clear. SGN degeneration is correlated with post-CI fibrosis and trauma and poorer hearing outcomes. (Nadol 2014, Pfingst et al., 2015; Schvartz-Leyzac et al., 2020) Elucidating the impact of macrophages on post-CI SGN health is relevant for development of more targeted strategies for selectively mitigating maladaptive aspects of the inflammatory response. As corticosteroids are nonspecific immunosuppressive agents, they might exert unwanted side effects and a more specific immunosuppressive agent might be more beneficial in this context.

In summary, our study suggests that macrophages (mononuclear phagocytes) play an important role in the intracochlear tissue remodeling that occurs following CI and in SGN health. Depletion of macrophages with PLX5622 reduces cellular infiltration into the scala media, but not fibrosis, following cochlear implantation. Moreover, macrophages appear to modulate the dynamics of fibrosis that contributes to increases in electrode impedances. Depleting a specific subset of mononuclear phagocytes (e.g., dendritic cells), lymphocytes, or non-immune cells will provide valuable information about their role in the post-CI FBR and inform translational efforts to mitigate this response. The current study describes the unique role of macrophages in cellular infiltration, fibrosis and SGN health following implantation. Further work is needed to understand the interplay of other immunologic cells following cochlear implantation that, along with macrophages, contribute to post-CI cochlear inflammation and FBR.

## Data Availability

Data supporting and validating the findings of this study are available within this article and its supplementary materials.
